# Etiology of Femoroacetabular Impingement in Athletes: A Review of Recent Findings

**DOI:** 10.1007/s40279-015-0339-2

**Published:** 2015-05-22

**Authors:** Amir A. Zadpoor

**Affiliations:** Department of Biomechanical Engineering, Delft University of Technology (TU Delft), Mekelweg 2, Delft, 2628 CD The Netherlands

## Abstract

The relationship between hip deformities and osteoarthritis has recently received a lot of attention. In particular, it has been shown that both osteoarthritis and its precursors, such as the hip deformities that lead to femoroacetabular impingement (FAI), are more prevalent in elite athletes compared with the general population. However, the etiology of the above-mentioned types of hip deformity is not currently well understood. Many recent studies have attempted to shed light on the etiology of this disease. In this article, the main clinical, radiological, mechanobiological, and biomechanical findings of relevance to understanding the etiology of hip deformities leading to FAI are reviewed. Based on these findings, a consistent biomechanical theory explaining the development of hip deformities in athletes is then presented. According to the presented theory, the repetitive, impact-like musculoskeletal loads that athletes experience, particularly when they undertake extreme ranges of hip motion, cause the development of hip deformities. According to this theory, these musculoskeletal loads trigger abnormal growth patterns during the years of skeletal development and cause the formation of hip deformities. A number of hypotheses based on the proposed theory are then formulated that could be tested in future studies to ascertain whether the proposed theory could sufficiently describe the development of hip deformities in athletes.

## Key Points

**Table:** 

The prevalence of cam-type deformity is higher within athletes compared with control groups.
Some of the data available in the literature suggest that the repetitive high-magnitude loads experienced by the athlete during years of skeletal development may contribute to the development of cam-type deformity.

## Introduction

Understanding the etiology of osteoarthritis has recently been the center of attention of many researchers. In particular, interest in the link between hip deformities and osteoarthritis that has previously received only limited attention [1–3], has recently revived [4] and the topic is currently being very intensively studied [5, 6]. Osteoarthritis is a multi-faceted, multi-organ disease that could develop due to a multitude of reasons. In many cases, no specific cause for the development of osteoarthritis can be identified—the so-called idiopathic osteoarthritis. Ganz et al. [4, 7] proposed a mechanical theory to explain the cause of some of the cases of osteoarthritis that were previously considered idiopathic. According to that theory, relatively minor developmental deformities such as femoroacetabular impingement (FAI) may lead to repetitive mechanical loading of cartilage and progressive damage that ultimately gives rise to osteoarthritis.

FAI is one of the few cases where the etiology of osteoarthritis and the associated risk for inducing cartilage damage have been extensively studied and documented [8–10]. FAI that is often subdivided into cam-type and pincer-type impingement results from abnormal anatomy of femoral head (cam-type) or acetabulum (pincer-type). There is also a mixed type of FAI in which both cam- and pincer-type deformities are found in the same hip. The overgrown parts of one bone may then impinge into the articular surface of the other when the individual undertakes extreme ranges of hip motion. The link between overgrowth anatomy and cartilage damage is therefore rather direct and mechanical.

Impingement of overgrown bone into the articular surface is one of the latest events in the development of osteoarthritis in FAI patients. A potentially more important question is ‘how does the abnormal anatomy of the femur or acetabulum develop?’ In the general population, there is some evidence that genetics may play a role in the development of hip deformities [11]. Any number of as yet unknown reasons may also contribute to the development of hip deformities. Understanding the etiology of FAI could have an important clinical and economic impact, particularly if it turns out that the mechanisms through which these deformities develop involve modifiable risk factors. Through modifying those potentially modifiable risk factors, one may be able to reduce the incidence rate of osteoarthritis within a certain group of individuals, thereby decreasing the high societal and material costs that are associated with the treatment of osteoarthritis. One specifically interesting case is the case of FAI in individuals routinely performing vigorous physical activities, such as professional and semiprofessional athletes [12]. As we will see in the next section, the prevalence of FAI [13–15], as well as osteoarthritis [16], is greater in the intensively training athletic population compared with the control groups composed of asymptomatic individuals from the general population or amateur players. During the last few years, a large number of researchers have studied FAI in different types of athletic populations, and similar patterns of prevalence have been found for various types of physical activities. It has therefore been hypothesized that there is a link between the vigorous physical activity undertaken by those individuals and the development of (specific types of) hip deformities [17]. The appropriate contexts for explaining any such relationship are biomechanics and mechanobiology. In addition to clinical and radiological studies, a number of mechanobiological and biomechanical studies have also been recently conducted to explain the development of FAI in the athletic population. The current article aims to (1) review both clinical and radiological findings (Sect. 2), as well as biomechanical and mechanobiological findings (Sect. 3) regarding FAI in the athletic population; and (2) study possible causal links between the biomechanical and mechanobiological factors and the development of hip deformities leading to FAI (Sect. 4). The main focus of this article is cam-type deformities, however pincer-type deformities are also discussed whenever possible.

## Clinical and Radiological Findings

The most important clinical and radiological findings are summarized in this section, with emphasis on the observations that are potentially important for understanding the etiology of the disease. In clinical settings, physical examinations including impingement tests are used to diagnose FAI and determine the range of hip motion [12]. Examples of clinical impingement tests include forced abduction, flexion, and internal rotation tests that are performed to determine whether these forced movements could elicit the symptoms of FAI [12].

Before presenting the radiological findings, it is important to discuss some of the most important concepts used in the radiological analysis of hip deformities. Alpha angle described by Nötzli et al. [18] is often used to assess the sphericity of the femoral heads seen on radiographs. A higher alpha angle is assumed to present a more severe cam-type deformity. In anterior–posterior radiographs, the alpha angle is measured by first fitting a circle to the femoral head and drawing a line that connects the center of the fitted circle to the center of the femoral neck [19]. A second line is then drawn from the center of the fitted circle to the first point of the superior surface of the head–neck junction that departs from the circle [19]. The angle between both lines is known as the alpha angle. In a recent study, cut-off alpha angles of 60° and 78° were found to define the presence of cam-type deformity and a pathological FAI condition, respectively [19]. However, other values of threshold are used in other studies; for example, see Barton et al. [20]. Magnetic resonance imaging (MRI) is also used for the diagnosis of FAI [10]. Alpha angle could also be measured using MRI images [20, 21]. For example, alpha angles have been previously measured on oblique axial and radial planes using MRI images [20, 21].

Center-edge angle (CEA) is used for the diagnosis of pincer-type FAI [22] and generally measures the acetabular coverage of the femur. In anterior–posterior radiographs, the lateral CEA is measured as the angle between the two following lines: a vertical line drawn from the center of the circle fitted to the femoral head, and the line connecting the lateral rim to the center of the circle fitted to the femoral head [23]. Large lateral CEA angles indicate over-coverage of the femur.

### Prevalence of Femoroacetabular Impingement (FAI) in Athletic Populations

Several studies have compared the prevalence of hip deformities in the preprofessional, semiprofessional, and elite athletic population with control groups often comprised of healthy individuals or amateur players [13, 15, 24]. These studies show that the radiological and clinical signs of FAI are more prevalent in athletic populations compared with control groups [13, 15, 24]. The rest of this subsection presents the details of these comparative studies for athletes exercising various types of sporting activities.

The prevalence of cam and pincer types of deformity measured using different radiographic parameters (including alpha angle) was 70 and 50 %, respectively, among elite male and female soccer players [25]. In a study of adolescent and young male soccer players, the presence of cam-type deformities was measured using a 3-point scoring system. A higher prevalence of anterosuperior flattening and anterosuperior prominence was found in the athlete group compared with the control group [13]. Moreover, the prevalence of increased alpha angle tended to be higher in the athlete group: 26 % of athletes vs. 17 % of the control group (*p* = 0.31) [13]. The range of motion of hips with cam-type deformity (alpha angle >60°) was lower than hips without cam-type deformity. Another comparative study of asymptomatic semiprofessional and amateur soccer players showed significantly higher values of alpha angle for the kicking leg of the semiprofessional group compared with the control group [15]. In addition, 22 % (5/22) of the semiprofessional players had positive clinical signs, while no amateur player exhibited any positive clinical findings [15].

In a sample of players in the National Football League with a history of hip pain or groin injury, 94 % (116/123) of the hips had radiographic signs of FAI, i.e. elevated alpha angle or decreased head–neck offset ratio [26]. In a similar study, 87 % of hip radiographs originating from the National Football League players (a mixed symptomatic and asymptomatic population) showed at least one radiographic sign of FAI [27]. Among all considered radiographic signs, only elevated alpha angle could predict groin pain [27]. Elite ice hockey players were also found to have significantly higher mean alpha angle values compared with a control group [24]. However, no difference in clinical findings was observed between the groups. None of the control group members and only one of the athletes had a positive impingement test result [24]. In a mixed population of symptomatic and asymptomatic capoeira players (a Brazilian marital art that requires extreme hip motions associated with kicking and jumping), different signs of hip deformity, including alpha angle, head–neck offset, crossover sign, acetabular index, lateral CEA, and the Tönnis grade, were assessed [28]. It was found that 92 % (44/48) of hips exhibited at least one radiographic sign of cam impingement [28]. A similar observation was made for track and field athletes; the mean alpha angle of the athlete group (44 participants) was significantly higher than the control group [14]. Moreover, seven of the track and field athletes had pathological signs, while no individual from the control group showed any signs of pathology [14].

### Relationship Between Type and Intensity of Physical Activity and FAI

Only limited information is available in the literature regarding the effects of the type and intensity of physical activity on the development of FAI. In one study, ice hockey players were found to be 4.5-fold more likely to show radiological signs of cam-type FAI, particularly elevated alpha angles, compared with skiers [29]. In a study of semiprofessional and amateur soccer players, a positive correlation between the number of training sessions per week and alpha angle was found [15]. A recent study [30] compared the incidence of cam-type deformity, defined as alpha angle >60°, between two groups of elite soccer players who had trained with different frequencies in their years of skeletal development. The prevalence of cam-type deformity was significantly higher in the group that trained four or more times per week compared with the group that trained three or less times per week [30].

### Development of Hip Deformities with Age

Studies that investigate the relationship between skeletal development and hip deformities have generally made the following three observations. First, it has been observed that hip deformities start to develop at a very early age, e.g. 10–12 years [13]. Second, the markers of hip deformity such as alpha angle tend to increase with age [29, 31, 32] and, finally, the development of hip deformity does not seem to occur once the physis is closed and the skeleton is mature [31].

Alpha angles exceeding 60° were found for some preprofessional soccer players, as well as some control group members, as early as 12 years of age [13]. In a follow-up study of young male soccer players, the prevalence of cam-type deformity increased from 2 to 18 % in hips with open growth plates [31]. However, there was no significant increase in the prevalence or severity of cam-type deformity in hips with a closed growth plate [31].

A positive correlation between age and alpha angle was observed for ice hockey players but not for the control group [29]. For ice hockey players, athletes with closed physes had significantly higher values of alpha angle compared with athletes with open physes [33].

In a computed tomography (CT)-based study of a population of 225 pediatric and adolescent individuals, the alpha angle was found to increase with age [32]. Moreover, the development of cam- and pincer-type deformities occurred at a very early age, i.e. 10–12 years [32].

### The Side of Hip Deformity

The presence or absence of symmetry in the development of FAI could give us some clues regarding the potential causes of such deformities in the athletic population. When a population of 22 asymptomatic semiprofessional soccer players was compared with a control group of amateur soccer players, it was found that semiprofessional players have a higher prevalence of elevated alpha angle in their kicking leg compared with the control group [15]. In the semiprofessional group, the kicking leg of 19 out of 22 players was the right leg. There was no significant difference between the prevalence of elevated alpha angle of semiprofessional and amateur players when only the left leg was considered [15]. In a study of asymptomatic female soccer players, professional players were found to have less internal rotation for their preferred kicking leg compared with non-professional players [34]. However, in two other studies, the prevalence of cam-type deformity was found to be similar between the dominant and non-dominant legs of soccer players [30, 31], leading researchers to suggest that movements other than kicking may be contributing to the development of cam-type deformity in these athletes [31].

In a population of elite soccer players, the prevalence of radiographic cam lesions in men was 68 % (51/75), of which 76 % were bilateral [25]. As for women, 50 % (10/20) had radiographic signs of cam lesion, of which 90 % (9/10) were bilateral [25].

It is important to note that FAI patients undergoing surgery often require bilateral surgery [35]. In general, male sex, younger age, higher alpha angle, and reduced acetabular anteversion at initial presentation were found to be significant risk factors for patients who ultimately required bilateral surgery [35]. Similar data are not available for the athletic population, and it is not clear to what extent the data presented for the general population extend to the athletic population.

### Sex-Specific Issues

The differences between male and female populations in terms of hip deformities have been studied by a number of researchers. In a study of former youth soccer players, the prevalence of cam deformity was found to be higher in men compared with women [36]. Moreover, elevated alpha angles were found to be more common among male elite soccer players compared with female elite soccer players [25]. Similar trends were found for the general population. A study of a generally young Swiss population showed that cam-type deformities are rare within the female population [37]; however, a higher prevalence of increased acetabular depth was found in the same population [37]. Another study of asymptomatic volunteers showed higher prevalence of cam-type deformity within men compared with women [38].

## Mechanobiological, Biomechanical, and Functional Findings

The biomechanical and mechanobiological findings regarding the FAI are reviewed in this section. The focus is on the studies and findings that could be used to understand the etiology of FAI.

### Mechanobiological Findings

**Fig. 1:**
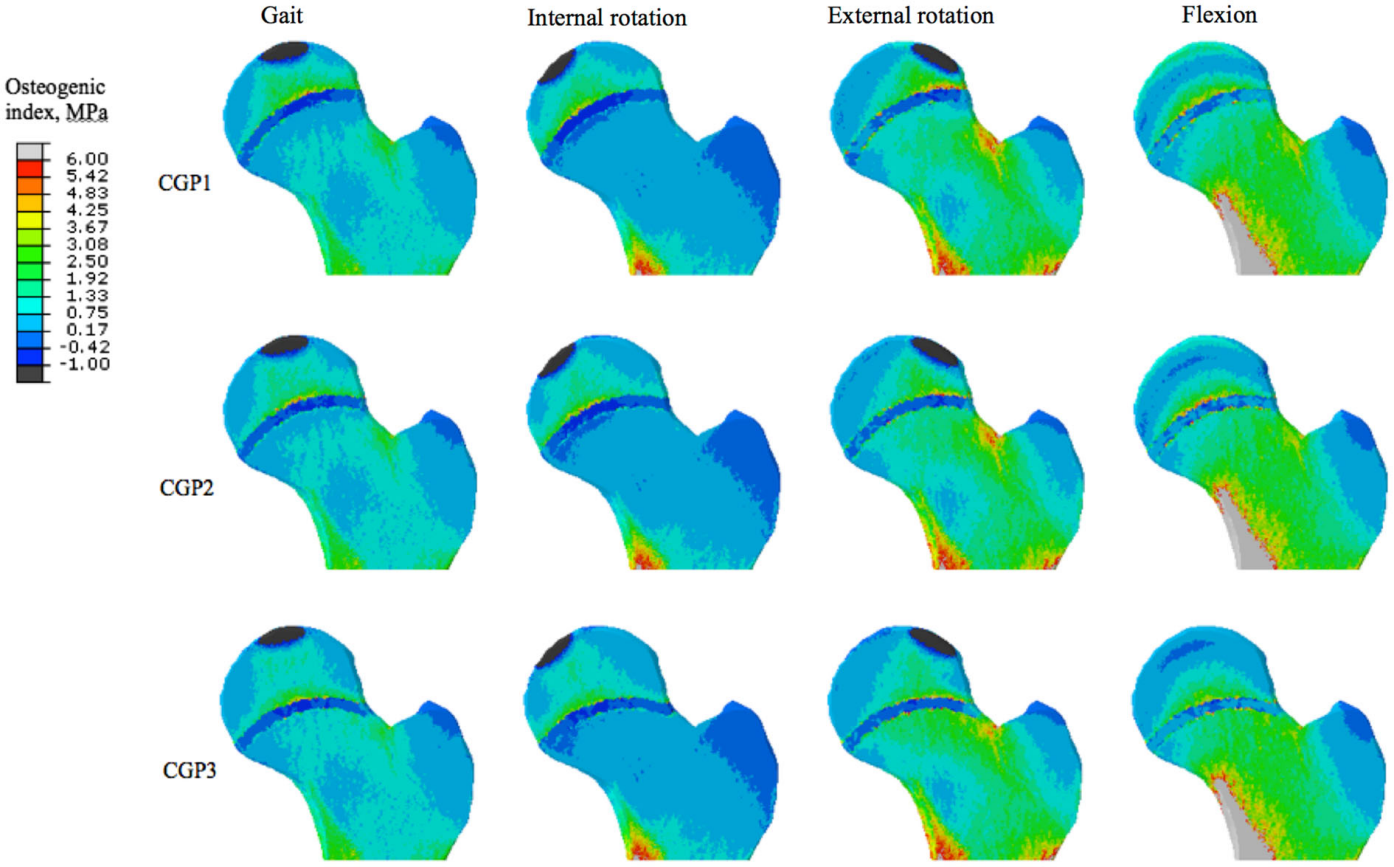
Distribution of the osteogenic index in the femur for different types of physical activity and different extension of the growth plate towards the femoral neck (CGP 1 to CGP 3 have progressively larger extensions towards the femoral neck) [17]. *CGP* curved growth plate. Reprinted from Roels et al. [17], with permission from the Osteoarthritis Research Society International. © 2014

**Fig. 2:**
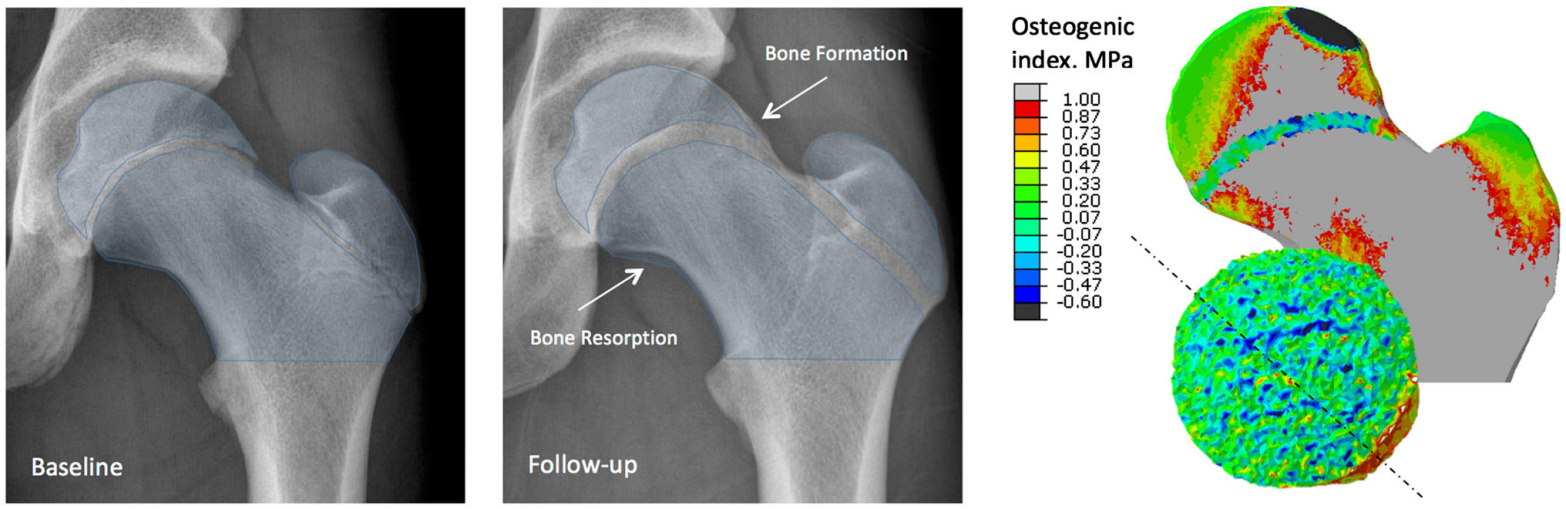
An X-ray of a representative young soccer player at baseline and follow-up, together with the distribution of the osteogenic index close to the growth plate. As the femur grows, there are areas of bone formation, and possibly even areas of bone resorption. These observations nicely explain load-driven development of a cam-type deformity. Reprinted from Roels et al. [17], with permission from the Osteoarthritis Research Society International. © 2014

Mechanobiological studies of the etiology of hip deformities in general, and FAI in particular, are rare. In theory, it is possible to use theoretical models of tissue growth and adaptation [39, 40] and patient-specific finite element (FE) models [41] to study how certain patterns of loading could influence skeletal development. However, not many studies have investigated these kinds of relationships with the aim of explaining the association between specific types of physical activity and the development of FAI. In a recent study [17], we considered the musculoskeletal loads associated with four different types of movements, namely gait, internal rotation, external rotation, and flexion, as well as different levels of growth plate extension towards the femoral neck. For every case, the mechanically-induced growth stimulus was calculated using FE models using the osteogenic index (OI) introduced by Carter and co-workers [42–47]. The OI has often been used to explain ossification patterns, including those of cartilage-like tissues [42] such as those seen in the growth plates of femora during skeletal development. In general, a higher OI means higher mechanical stimulus for bone growth. The FE model was based on the CT images of a 12-year-old individual and included the growth plate shape and properties. The results of that study showed the strong influence of the type of physical activity and growth plate extension towards the femoral neck on the OI distribution (Fig. 1). As the growth plate extension towards the femoral neck increased, the OI values on the proximal and distal sides of the growth plate increasingly deviated from each other (Fig. 1). The OI values were generally higher for external rotation and flexion compared with gait and internal rotation. Moreover, in external rotation and flexion, the unbalance between the OI values on both sides of the growth plate occurred close to the area where cam-type deformities usually develop (Fig. 2). These two last observations indicate that not all activities, but rather specific types of physical activity, increase the chance of developing cam-type deformity.

### Functional and Biomechanical Findings

Musculoskeletal loading is directly related to the physical activities undertaken by individuals. In order to study the musculoskeletal loading of athletes, one usually needs to study the movement of their different body segments and the forces exerted to their body during those movements (e.g. the ground reaction force). One could then relate those to internal musculoskeletal forces, including muscle and joint reaction forces, using biomechanical models such as musculoskeletal models [48, 49] or mass-spring-damper models [50, 51]. Biomechanical studies of athletes in relation to FAI could be performed in two contexts. First, one could study athletes’ movement patterns and musculoskeletal forces while they perform specific sport-related physical activities that involve extreme ranges of motion and repetitive impact. The same type of analysis could be performed for normal physical activities such as gait. Moreover, both types of the above-mentioned analyses could be performed for symptomatic and asymptomatic athletes. Indeed, these types of analysis may need to be performed separately for individuals who are at different stages of FAI development, from inception of hip deformity to advanced stages of cartilage lesions.

These types of analysis are only scarcely available in the literature. Perhaps the most well-studied case is the case of normal physical activity of symptomatic FAI patients [52–56]; however, these studies are not very useful in understanding the etiology of the disease. In a few studies [34, 57], some biomechanical and functional data are reported for asymptomatic athletes in normal physical activities such as gait, and in clinical examinations. In particular, semiprofessional soccer players exhibited significantly higher loading rate, peak vertical force, and peak tibial acceleration compared with amateur soccer players [57]. In turn, the maximum rearfoot motion of the semiprofessional players group was found to be significantly smaller than amateur soccer players [57]. In another study, professional female soccer players were compared with non-professional players, and were found to exhibit significantly smaller flexion and internal rotation, respectively, for both their hips and preferred kicking leg [34]. More signs of the association between FAI and the limited range of hip motion, including internal rotation <10°, were found in a different study of 226 asymptomatic adolescent athletes [58].

There are nil to very limited biomechanical and functional data regarding the musculoskeletal loads experienced by symptomatic or asymptomatic athletes when performing sport-related physical activities at extremes ranges of hip motion and possibly involving repetitive impact loading. This type of information is the most valuable type of biomechanical data when studying the etiology of sport-related hip deformities.

## Possible Causal Relationships

The clinical, radiological, mechanobiological, and biomechanical findings reviewed earlier could be used to propose a theory for the development of hip deformities in athletes. Since the prevalence of cam-type deformity is greater in athletes compared with control groups (Sect. 2.1), it is reasonable to assume that physical activities somehow lead to the development of these hip deformities. On the other hand, the currently available evidence shows that the development of hip deformities starts at a very early age when the skeleton is not mature, that cam-type deformity does not develop after the closure of the growth plate, and that the alpha angle increases with age during adolescence (Sect. 2.3). It is therefore reasonable to assume that the development of cam-type deformity is related to skeletal development. In this scenario, the mechanical loads experienced during vigorous physical activity result in certain patterns of local stress in the growth plate and its surrounding areas. This is partly due to the mechanical properties of the cartilage-like tissue in the growth plate that are different from those of the surrounding bone. Since the above-mentioned loads are generated at the extreme ranges of hip motion, the joint reaction loads may be applied at the areas of the femur that do not normally experience large musculoskeletal loads. This could lead to generation of mechanical stimulus for bone growth in the areas of the femur that do not normally experience mechanical growth stimulus. The process of skeletal development therefore deviates from the usual case where the dominant mechanical loads of the femur are balanced, the extreme ranges of hip motion are rare, and the sphericity of the femoral head is preserved. This is consistent with the above-mentioned mechanobiological finding that specific types of physical activity, e.g. external rotation or flexion, stimulate the development of cam-type deformity and not the usual loading experienced in, for example, gait (Sect. 3.1). In addition to the type of physical activity, the intensity and frequency of physical activity could play a role in the development of hip deformities. It is known that a higher frequency of load application and application of greater mechanical loads could both lead to increased bone apposition [39, 59]. Moreover, the highly dynamic and impact-like nature of mechanical loading in certain sports could generate higher levels of mechanical growth stimulus. This is consistent with the above-mentioned findings which show that there is a positive correlation between alpha angle and training intensity (Sect. 2.2), that professional players have elevated alpha angles and higher prevalence of deformities compared with non-professional players who normally train less (Sect. 2.1), and that the prevalence of hip deformities may be dependent on the type of physical activity (Sect. 2.2). Biomechanical findings (Sect. 3.2) also show that the loading of the lower extremity, including the peak force and loading rate, are higher in asymptomatic semiprofessional players. This further strengthens the theory that the development of hip deformities in athletes is caused by load-driven deviations from the normal growth patterns during the years of skeletal development.

## Hypotheses for Future Research

According to the biomechanical theory laid out in the previous section, the high-magnitude, impact-like forces that athletes experience during their years of skeletal maturation could influence the development of the femoral anatomy and contribute to the development of cam-type deformity. In order to assess this biomechanical theory, one needs to develop falsifiable hypotheses that are based on this theory. In this section, we mention some of the hypotheses that have either been not studied before or have been studied only in passing in a few (possibly low-power) studies.

1. Development of cam-type deformity does not occur after the closure of growth plate or is significantly slowed down: As seen in Sect. 2.3, there is some evidence that the development of cam-type deformity occurs only for individuals with open growth plates [31]. However, more data are needed for establishing the validity of this hypothesis as it is one of the most important hypotheses regarding the etiology of FAI.
2. There is a positive correlation between training intensity and the radiographic/clinical sings of FAI: We have previously seen (Sect. 2.2) that there are already some data to support this hypothesis [15, 30]. However, much more data from high-power studies are needed to assess the validity of this hypothesis.
  One of the major challenges in assessing this hypothesis is in developing a unified measure of training intensity. As long as one specific type of sport is considered, it might be acceptable to measure training intensity by the number of training sessions per week or the total number of training hours per week, or similar measures. However, this type of measure will not work when comparing different types of sporting activities. An alternative approach for unifying the different types of sporting activities would be the use of more objective measures such as the magnitude, rate, and frequency of the loads experienced during the training sessions, together with the total training time. However, this will require specific measurement techniques that may prove difficult to implement in professional training settings. Even one step further would be the use of biomechanical models to translate the above-mentioned metrics to the loads experienced by the femur, and combining these with bone tissue growth models that could predict bone shape during skeletal development to better quantify the stimulus for bone growth.
3. The prevalence of cam-type deformity is dependent on the type of sport. Sports that involve extreme ranges of hip motion and high-impact movements have a higher prevalence of FAI markers: This hypothesis has not been thoroughly studied before and needs to be assessed in future studies.
4. In asymmetric sports where one leg is used more intensively, or in more extreme hip motions, the prevalence of FAI markers is higher in the more intensively used leg: A few studies did not find any evidence that the prevalence of FAI markers is higher in the dominant leg of soccer players [30, 31]. On the other hand, loads experienced during movements other than kicking may be contributing to the development of cam-type deformities [31]. Additional information from biomechanical studies could be useful in designing proper experiments and identifying the correct types of sporting activities for assessing the validity of this hypothesis. In particular, it is important to know what kind of musculoskeletal loads result from different types of physical activities and identify the type of musculoskeletal loads that could contribute to the development of cam-type deformity.

## Discussion

The etiology of the hip deformities predisposing individuals to FAI is not currently well understood; however, there has been intensive clinical, radiological, mechanobiological, and biomechanical research during the last few years. The findings of these research projects have provided us with a foundation of facts upon which a theory regarding the etiology of hip deformities in athletes could be built.

In this article, the currently available clinical, radiological, mechanobiological, and biomechanical findings relevant for the study of etiology of FAI were reviewed, and a theory consistent with these facts was presented as to how hip deformities develop in athletes. However, the level of evidence available in the literature is not enough to enable us to decisively accept or reject the presented theory. That is why it is of the utmost importance to test the hypotheses presented here (Sect. 5), as well as other relevant hypotheses, using high-power studies of different types of athletic populations. Moreover, the modulations of the presented theory with other pathways of cartilage damage, such as inflammation, should be carefully studied [60].

The hypotheses presented in the previous section are important for assessment of the validity of the presented theory. However, more could be learned about the potentially developmental nature of cam-type deformity through the study of the relationship between the movements of athletes during sporting activities, the musculoskeletal loads experienced by athletes when undertaking those movements, and the development of cam-type deformity. As previously mentioned, biomechanical techniques, such as motion capture systems, the apparatus for measurement of external forces, and inverse dynamic musculoskeletal models, could be used to understand the type, magnitude, and direction of musculoskeletal loads experienced by athletes when undertaking any given physical movement. On the other hand, the obtained musculoskeletal loads could be used in FE models that incorporate bone growth models to understand whether any given movement could contribute to the development of cam-type deformity by adversely affecting the mechanical bone growth stimulus. If we know which physical movements might contribute to imbalances in the bone growth stimulus, it might be possible to develop compensatory exercises that, although not necessarily needed for the training of players, could restore the balance of the mechanical bone growth stimulus, thereby ensuring that hip deformities do not develop.

Robust methodology is, in any case, very important for these endeavors. There are several technical details that are important regarding the methodology used in such investigative studies. In this paper, two specific technical points—one pertaining to clinical and radiological studies and the other pertaining to biomechanical and mechanobiological studies—are highlighted.

Regarding the radiological and clinical point, it is important to realize that controversy exists as to whether radiological signs of FAI are indicative of the actual disease [61, 62]. Radiological signs may also be quite common in healthy young individuals [63]. In fact, a study of a hospital population showed that radiological signs were only absent in 11 % (58/522) of the hips of patients who were not suspected of having FAI [64]; however, the alpha angle that is reported in many of the studies reviewed here was not included in this study. Since (radial) alpha angle is known to be one of the best predictors of FAI symptoms [65], caution needs to be exercised when interpreting the results of that study. In cases where the radiological signs are present but there are no symptoms of the disease, it is often assumed that the individual is ‘predisposed’ for FAI [15] but may not have developed the symptoms yet. However, this may not be true as the radiological signs of FAI seem to be non-specific [64]. In any case, accurate definitions of alpha angle thresholds [19] and reference values for the hip anatomy [66] are needed for a more precise radiographic analysis of hip deformities.

As for the biomechanical and mechanobiological point, an accurate description of the musculoskeletal loading during extreme ranges of motion and impact-intensive physical activities is currently lacking. The proper methodological approach will require the use of motion tracking systems, force plates or pressure-sensitive pads, and patient-specific musculoskeletal models to estimate the detailed loading, including muscle and joint reaction forces when performing physical activities. Due to the lack of such detailed information, mechanobiological studies on the development of hip deformities, for example the study by Roels et al. [17], will have to use subjectively estimated loads that may not be an accurate representation of the actual loading conditions experienced by athletes during these specific physical movements.

Ultimately, it is important to realize that the decreased range of hip motion in athletes may need to be compensated by increased pelvic motion, thereby subjecting pelvic stabilizers to higher stresses and possibly resulting in damage to the affected soft tissues [67]. FAI in athletes may therefore be associated with secondary conditions resulting from those compensatory mechanisms. Future studies are needed to clarify the role of neuromuscular compensatory mechanisms in (co-) development of FAI and any associated secondary condition.

As described by Pun et al. [68], and found in a recent systematic review of the literature [69], non-surgical treatments such as physical therapy and modification of activity could be beneficial for patients. There is also a range of surgical treatment options [70] that could be pursued if the conservative treatments fail to deliver the desired outcomes. Exploring different conservative treatments based on exercises and physical therapy constitutes another worthwhile avenue for future research.

## Conclusions

The clinical, radiological, mechanobiological, and biomechanical findings seem to hint towards a possible theory for the development of hip deformities in athletes. According to this theory, these types of deformities are caused by abnormal growth during the years of skeletal development that are stimulated by repetitive impact-like musculoskeletal loads experienced by athletes when they perform physical activities involving extreme ranges of hip motion. However, the currently available evidence is not decisive and more high-power studies focused on specific hypotheses predicted by the above-mentioned theory are needed.
